# Isolation, characterization and biological activities of betulin from *Acacia nilotica* bark

**DOI:** 10.1038/s41598-022-13338-3

**Published:** 2022-06-07

**Authors:** Prabhjit Kaur, Saroj Arora, Rajbir Singh

**Affiliations:** 1 Department of Botanical and Environmental Sciences, Guru Nanak Dev University, Amritsar, Punjab 143001 India; 2 Medicinal Plant Research Laboratory, Post Graduate Department of Botany, Khalsa College, Amritsar, Punjab 143001 India

**Keywords:** Biological techniques, Cancer, Drug discovery

## Abstract

Medicinal plants are in use of humankind since ancient and still they are playing an important role in effective and safer natural drug delivery systems. *Acacia nilotica* (native of Egypt) commonly known as babul belongs to family *Fabaceae,* widely spread in India, Sri Lanka and Sudan. Being a common and important plant, using in many ways from fodder (shoots and leaves to animals) to dyeing (leather coloration) to medicine (root, bark, leaves, flower, gum, pods). The present study is focused on investigating the natural chemistry and important biological activities of the plant. Employing bioassay guided fractionation coupled with TLC and column chromatography, a pure fraction named AN-10 was isolated from ethyl acetate fraction of crude methanol extract which identified as “Betulin (Lupan-3ß,28-diol)” by Liebermann-Burchard test and structure elucidation by UV–Vis, NMR and MS techniques. A battery of in vitro biological assays for antioxidant, anti-inflammatory and anticancer were performed and betulin showed excellent potential in all assays. It was found that the inhibitory potential in all assays were dose dependent manner and after a range of concentration, the activities get leveled off with no further increase in activity.

## Introduction

Increasing evidence from epidemiological and biological studies has shown that reactive oxygen species (ROS) are involved in variety of physiological and pathological processes^1,2^. Plant and food derived antioxidants are implicated in the prevention of cancer and aging by destroying oxidative species that initiate carcinogenesis through oxidative damage of DNA^3,4^. Previous scientific reports confirmed an inverse association between the daily consumption of fresh fruits & green vegetables and the chances of degenerative & chronic diseases^5^. The phenolic compounds of fruits and vegetable act as antioxidant through various ways, which includes complexation of redox-catalytic metal ions, scavenging of free radicals, and decomposition of peroxides. Especially in food-related systems (extracts/fractions), antioxidant activity studies using multiple experimental approaches, allow a complete screening of the putative chain-breaking capacity^6^. The phenols and polyphenols have attracted the interest of medical scientist because of their pharmacological properties^5,7^. *Acacia nilotica* (L.) Willd. Ex Del., (family *Fabaceae*) is a medicinal tree known for the versatile source of bioactive components. This plant offers a variety of compounds which are potent for their spasmogenic, vasoconstrictor, anti-hypertensive, antioxidant, antispasmodic, anti-inflammatory and anti-platelet aggregatory properties^8^. The leaves & flowers of *A. nilotica*, an evergreen tree are also been used as animal fodder^9–11^. The bark of the plant is rich with condensed tannins, catechin, epicatechin, epigallocatechin gallate and has also been used for the treatment of viral, bacterial, amoeboid, fungal, bleeding piles & leucodermal diseases^12^. The previous studies performed at Genetic Toxicology Laboratory of GNDU has shown that bark of *A. nilotica* enriched with kaempferol, umbelliferon, gallic acid, ellagic acid, which are responsible for their potent antioxidant, antimutagenic and cytotoxic activities^8,9,13^. The lack of detailed & systematic phenolic profiling of *A. nilotica*, which might be responsible for their important biological activities, led us to design the present study. In this study, HPLC based phenolic fingerprinting, bioassay guided fractionation, isolation & identification of betulin (AN-10) from ethyl acetate fraction of crude methanol extract of *A. nilotica* was done. The betulin was further checked for their antioxidant activities (DPPH, Deoxyribose, Chelating power, reducing power, lipid peroxidation assays), cytotoxic (SRB assay) & anti-inflammatory activities (COX-2 inhibitory assay).

## Methods

### Chemicals

2’-2’ Diphenyl-1-picrylhydrazyl (DPPH) and Betulin (Lupan-3ß,28-diol) were obtained from Sigma Chemical Co. (St. Louis, MO, USA) and 2-deoxyribose was obtained from Lancaster Synthesis Inc. (Windham, USA). Adriamycin, 5- Fluorouracil (5-FU), Mitomycin-C, Trypsin, RPMI-1640 medium, acetic acid, trichloro acetic acid (TCA), fetal calf serum (FCS), gentamycin, penicillin, and 2-Thiobarbituric acid and HPLC authentic standards were purchased from Sigma–Aldrich USA. Human Cancer Cell lines were procured from National Cancer Research Institute, USA. Sulforhodamine B from Fluka, phosphate buffer saline (PBS) from Merck (Germany) and Tris EDTA from Hi Media. All stock solutions were prepared in double distilled H_2_O. All other chemicals were of analytical grade and procured from Ranbaxy Fine Chemicals Ltd. (New Delhi, India). The anti-inflammatory bioassay kit was purchased from Cayman Chemicals (Michigan, USA).

### Collection and identification of plant material

The bark material of *A. nilotica* was collected in the month of November from a tree grown at the front side of Bebe Nanaki Girls Hostel-II, Guru Nanak Dev University (GNDU), Amritsar (As per permission and guidelines from competent authority). GNDU is located at 31.6340° N, 74.8259° E with loamy soil texture. Plant identification was conducted at the herbarium in the Department of Botanical & Environmental Sciences, GNDU, Amritsar–India, where a voucher specimen of *A. nilotica* is deposited (A/C # 6421, dated 12-01-2007). All plant experiments were performed in accordance with relevant guidelines and regulations of institution.

### Sample preparation and extraction

**Figure 1:**
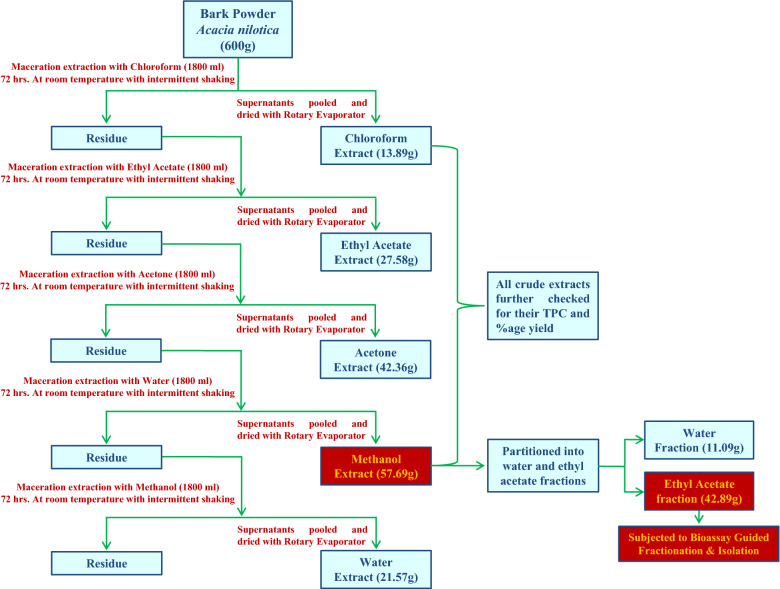
Extraction/fraction procedure of Methanol and other crude extracts from bark powder of *Acacia nilotica.*

The bark material was washed with tap water (thrice) to remove dust particles, dried in oven at 40 °C for 24 h and grounded to fine powder. The fine bark powdered material (600 g) of *A. nilotica* was macerated first with chloroform (1800 ml) for 72 h with intermittent vigorous shaking and after every 24 h supernatant was filtered, and the dried powder was re-macerated twice with fresh chloroform solvent. Then all supernatants pooled and dried by using a rotary evaporator (BUCHI R-300, SWITZERLAND). The crude methanol extract, which was used in present study, was obtained after maceration extracting the bark powder in chloroform, ethyl acetate and acetone. i.e. increasing order of solvent polarity. The methanol extract was further fractioned into water and ethyl acetate fractions (Fig. 1). The dried crude extracts were transferred into vials and kept in a desiccator until use.

### Determination of total phenolic content

The Total Phenolic Content (TPC) of different crude extracts of *A. nilotica* was determined by the method of Folin Ciocalteu^14^ as gallic acid equivalent (GAE) in milligram per gram extract sample.

### HPLC analysis of ethyl acetate fraction of methanol extract

Standard stock solution of gallic acid, quercetin, myricetin, rutin, quercetin, kaempferol, catechin, epicatechin, ferulic acid and 7-hydroxycoumarin were prepared as 1 mg/1 ml in HPLC grade methanol: water (90:10). HPLC analysis was performed on a Shimadzu Prominence HPLC system and Shimadzu LC solution (ver. 1.21 SP1) software. Chromatography was carried out on a Luna C18 (2) column (250 mm × 4.6 mm, 5 μm particle size). At a column temperature of 27 °C and a flow rate of 0.80 mL/min using solvent A (water) and solvent B (0.02% trifluroacetic acid (TFA) in acetonitrile) with a linear gradient elution: 70% A (5 min), 15–35% (7 min), 35–45% (11 min), 45–35% (16 min), 35–15% (20 min) at λ 280 nm. Stock solution containing ten analytes were prepared and diluted to appropriate concentrations for establishing calibration curves and different concentrations of theses analytes were injected thrice for the quantitative analysis and the calibration curves were constructed by plotting the peak areas versus the concentration of each analyte. The selectivity of the method was determined by analyzing standards and methanol extract. The peaks of reference compounds were identified by comparing their retention times (rt in min.) with the spectrum of authentic standard (Sigma Aldrich, USA).

### Bioassay guided fractionation and isolation of triterpenoids

In the process of bioassay-guided fractionation, ethyl acetate fraction of crude methanol extract is first tested for their activities, then fractionation and separation through column and TLC and then the resulting fractions are again tested for activity. The most active fractions (#56–154) with similar spot on pre-coated TLC plates is processed further for the separation of triterpenoids by column chromatography (data shown in results section).

**Figure 2:**
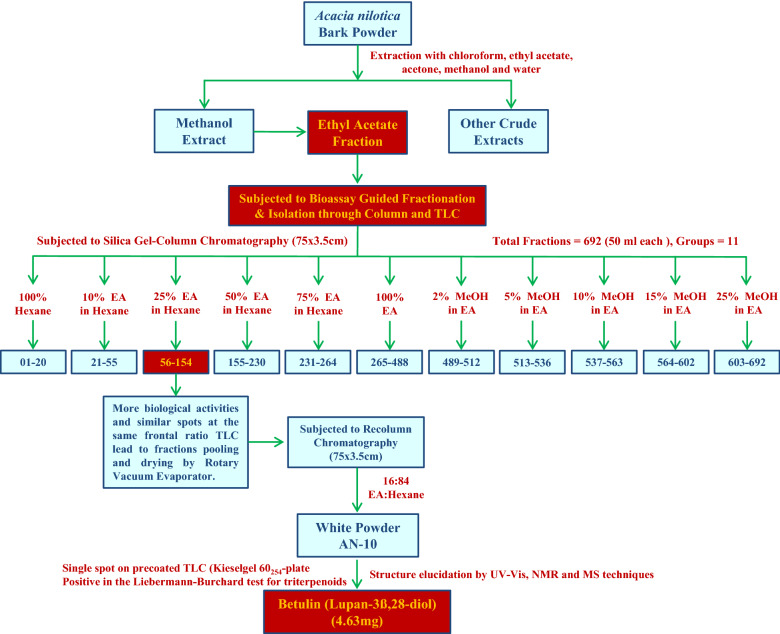
Bioassay guided fractionation and isolation of triterpenoids from ethyl acetate fraction of methanol extract of *Acacia nilotica* bark powder.

20 g ethyl acetate fraction of methanol extract mixed with celite ‘545’ was suspended in methanol and subjected to column chromatography using a 75 × 3.5 cm glass column filled with acidic alumina (brockman’s activity) upto 5 cm down from the top of glass column. After bedding down the silica gel, column elution stated with 100% hexane and then conducted by successive applications of solvent gradients of hexane/ethyl acetate 90;10, 75:25, 50:50, 25:75, 0:100 then with solvent gradient of methanol/ethyl acetate 2:98, 5:95, 10:90, 15:85, 20:80 to collect total of 692 fractions (50 ml each). Preliminary thin layer chromatography of all total 692 fractions was done to check number of compounds in each elution (based on *rf* values and no. of spots). Elutions showing same spots on TLC plates were pooled, concentrated and dried with Rotary evaporator to obtain high purity fractions. Fractions numbering 56–154 eluted ethyl acetate/hexane 25:75 showed single spot (same *rf* value) on pre-coated TLC plates lead to pooling and drying of fractions. The pooled and dried fractions were re-chromatographed with solvent gradient of ethyl acetate/hexane and fraction named “AN-10” eluted with 16;84 (ethyl acetate:hexane) resulted in isolation of a white 4.63 mg amorphous powder (Fig. 2).

### Identification of AN-10 fraction by NMR and MS techniques

^1^H and ^13^C NMR spectra were recorded for purified “AN-10” fraction at 300 MHz, using 5-mm sample tubes on a Bruker Avance-300 spectrometer. CD_3_OD was used as solvent for measurements at 30 °C. For structure elucidation and complete spectrum analysis, other additional experiments were performed as necessary: DEPT, ^13^C observation with selective ^1^H decoupling, 2D H,H-COSY. ^13^C chemical shifts δ are reported in ppm relative to TMS with an internal reference. With very few exceptions all NMR assignments are unequivocal. Mass spectra were recorded on QTOF-Micro of water Micromass. Melting point was determined on a Barnstead Electrothermal 9100.

### Antioxidant activities testing assays

In vitro antioxidant activities (AOA) of the crude extracts/fractions of *A. nilotica* and “AN-10” fraction was addressed by employing DPPH scavenging assay measured in terms of hydrogen using the stable nitrogen centered radical DPPH following the method of Blois^15^. The hydroxyl radical scavenging was checked with site specific and non-site specific deoxyribose degradation method of Halliwell et al.^16^ and Arouma et al.^17^. The reducing power was determined as described by Oyaizu^18^. The chelating effect on ferrous ions was determined according to the method of Dinis et al.^19^ and Lipid Peroxidation (LPO) was determined according to Halliwell & Guttridge^20^.

### In vitro cytotoxicity assay

The Sulforhodamine B dye assay was used for In vitro cytotoxic screening of crude extracts/fractions of *A. nilotica* and “AN-10” fraction according to Skehan et al.^21^. For primary screening, A-549 (Lung), DU-145 & PC-3 (prostate), IGROV-1 (Ovary) and MCF-7 (Breast) cancer cell lines were used. The treatments were (OD) was recorded at 540 nm, on ELISA reader and percent growth inhibition in the presence of extract/fraction and “AN-10” was calculated.

### Anti-inflammatory activity

In vitro COX-2 inhibiting activities of crude extracts/fractions of *A. nilotica* and “AN-10” fraction has been evaluated using ‘COX (ovine) inhibitor screening assay’ kit with 96-well plates. Both ovine COX-1 and COX-2 enzymes were included^22^. This screening assay directly measures PGF_2_α produced by SnCl_2_ reduction of COX-derived PGH_2_. The wells of the 96-well plate showing low absorption at 405 nm indicate the low level of prostaglandins in these wells and hence the less activity of the enzyme. Therefore, the COX inhibitory activities of the crude extracts/fractions of *A. nilotica* and “AN-10” fraction could be quantified from the absorption values of different wells the 96-well plate.

### Statistical analysis

All experimental analyses were performed in triplicate (n = 3) and the data was presented as mean ± SD on excel sheet. For in vitro antioxidant assays, one way ANOVA test followed by Tukey’s test (*P* < 0.05) was used to analyze the differences among IC_50_ of various AN-10 and extract/fractions for different antioxidant assays.

## Results

### % yield of extract, TPC and bioassay guided fractionation

**Figure 3:**
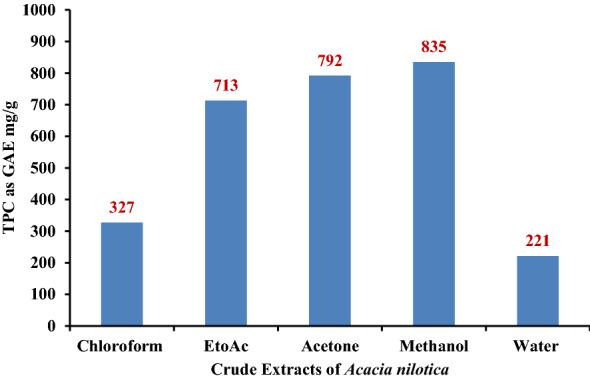
Total Phenolic Content of different crude extracts from bark of *Acacia nilotica* in mg/g as GAE (Gallic acid equivalent).

The high % yield (57.69 g and 6.15% yield) and Total Phenolic Content (835 mg/g as GAE) of methanol extract than other crude extracts of *A nilotica* lead for the detailed bioassay-guided fractionation (Fig. 3), HPLC based phytochemical screening and biological activities. Silica-gel column chromatography was performed on ethyl acetate fraction (42.89 g) of methanol extract of *A. nilotica* and 692 fractions of 50 ml each were collected. All these fractions were pooled into 11 groups according to their similar spot at the same frontal ratio on thin layer chromatography profiles and biological activities (Fig. 1). 54–156 fractions (group 3) exhibited high antioxidant, anti-inflammatory & anticancer activities as compared to other fractions and group of fractions (Table 1). In order for the detailed chemical investigation and identification of active compounds, the most active fractions (group-3) were pooled, dried and fractionated through re-column chromatography (silica gel, 75 × 3.5 cm) and “AN-10” fraction was collected by solvent gradient of ethyl acetate/hexane (16:84). Other chromatography and spectroscopy techniques were used for identification and structure establishment of “AN-10” fraction.

**Table 1:** Bioassay guided fractionation based biological activities of fractions collected from methanol extract of *Acacia nilotica* through column chromatography.

Group	Fraction numbers (50 ml each)	In vitro bioactivity testing assays							
		DPPH	Deoxyribose Degradation		Reducing power	Chelating power	Lipid peroxidation	COX	Cancer
			SS	NSS					
Group -1	01–20	** + + **	** + + **	** + + **	** + **	** + + **	** + + + **	** + + **	** + + + **
Group -2	21–55	** + + + **	** + + **	** + + **	** + + **	** + + **	** + + + **	** + + **	** + + **
Group -3	56–154	** + + + + **	** + + + + **	** + + + **	** + + + + **	** + + + **	** + + + + **	** + + + + **	** + + + + **
Group -4	155–230	** + + **	** + **	** + + **	** + + + **	** + + **	** + + + **	** + + **	** + + + + **
Group -5	231–264	** + + **	** + + **	** + **	** + + **	** + + **	** + + **	** + + + + **	** + + **
Group -6	265–488	** + + + **	** + + + **	** + + **	** + + + **	** + + + + **	** + + + **	** + + + **	** + + + **
Group -7	489–512	** + + + **	** + + **	** + + + **	** + + **	** + + **	** + + + **	** + + **	** + + + **
Group -8	513–536	** + + + + **	** + + + **	** + + **	** + + + **	** + + + **	** + + + **	** + + + + **	** + + + **
Group -9	537–563	** + + + + **	** + + + **	** + + **	** + + + **	** + + + **	** + + **	** + + + **	** + + + + **
Group -10	564–602	** + + + **	** + + + **	** + + + **	** + + **	** + + **	** + + + **	** + + + **	** + + + **
Group -11	603–692	** + + + **	** + + **	** + + **	** + + **	** + **	** + **	** + + **	** + + **

SS: Deoxyribose Site specific assay; NSS: Deoxyribose non-site specific assay.

Activity percentage (%) range: + : 0–25%, +  + : 25–50%, +  +  + : 50–75%, +  +  +  + : 75–100%.

In HPLC analysis, for the better resolution, different mobile phases were used and after several trails, mobile phase consisting of solvent A (water) and solvent B (0.02% trifluroacetic acid (TFA) in acetonitrile) as a solvent gradient was finely selected in order to achieve optimal separation & quantification, high sensitivity, and good peak shape. Table 2 shows the Retention Time (RT in minutes) and % quantification as µg/mg of 10 major polyphenols.

**Table 2:** HPLC based phenolic fingerprinting and quantification of the major polyphenols in ethyl acetate fraction of crude methanol extract of *Acacia nilotica*.

Compound	RT (min)	Quantification (μg/mg)	Molecular formula	Molecular mass
Gallic acid	3.57	156.26	C_7_H_6_O_5_	170.12
Catechin	4.83	265.19	C_15_H_14_O	290.27
Epicatechin	5.61	196.35	C_15_H_14_O	290.27
Rutin	7.10	109.78	C_27_H_30_O_16_	610.52
Umbelliferone	9.24	183.17	C_9_H_6_O_3_	162.14
*o*-Coumaric	10.68	t	C_9_H_8_O_3_	164.16
Quercetin	12.95	161.91	C_15_H_10_O_7_	302.24
Myricetin	11.09	117.29	C_15_H_10_O_8_	318.24
Betulin	15.42	56.83	C_30_H_50_O_2_	442.72
Kaempferol	16.10	19.27	C_15_H_10_O_6_	286.24

RT: Retention Time.

“t” indicates “trace”.

The presence of these polyphenols, in methanol extract of *A. nilotica*, was confirmed by comparison of their retention times and overlaying of UV spectra with authentic standards. The methanol extract which showed presence of these 10 polyphenols, among which catechin, epicatechin, quercetin gallic acid, umbelliferone, rutin and myricetin quantitatively found in considerable amount while Kaempferol & betulin were found present in traces amount whereas *o*-Coumaric was not detected. Many unknown peaks were also observed in the chromatogram which was characterized as the glycosides of flavonols.

### Identification and structure elucidation of “AN-10” fraction

**Figure 4:**
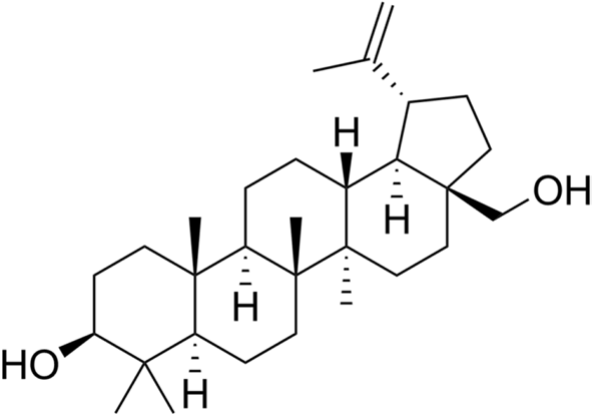
Chemical structure of AN-10 fraction (C_30_H_50_O_2_).

The results of the present study showed that the methanol extract of *A. nilotica* contains a complex mixture of polyphenols consisting mainly of polyhydroxyflavan-3-ols (catechins, epicatechin) and ellagic acid derivatives. The chromatographic purification of this extract resulted in the isolation of compound AN-10 (4.63 mg). These results provided unequivocal determination of structures and stereochemistry. The key evidence and arguments used to define the structures shown are briefly described. Fraction “AN-10” isolated as a white amorphous powder which were positive in the Liebermann-Burchard test for triterpenoids. Its positive ion HRESI-QTOF-MS displayed protonated molecular ion peak [M^+^H]^+^ at m/z 443 corresponded to the molecular formula C_30_H_50_O_2_. The ^1^H NMR spectrum of AN-10 indicated the presence of six methyl groups at H 0.76 (s, H3-24), 0.82 (s, H3-25), 0.97 (s, H3-23 and H3-27), 1.02 (s, H3-26), 1.68 (s, H3-30) together with two diastereotopic protons for a methylene group attached to hydroxyl at H 3.31 and 3.78 (d, J = 10.7, H28 and H-28') and two exocyclic methylene protons at H 4.58, 4.68 (s, H-29 and 29') established lupane skeleton for compound^23^. ^13^C NMR spectrum displayed signals due to six methyl carbons at C 14.9, 15.5, 16.1, 16.2, 19.2 and 28.1, oxygen-bearing methine and methylene carbons at C 79.1 and 60.7 and a set of exocyclic olefinic carbons at C 109.6 and 150.6. The ^1^H and ^13^C NMR signals for exocyclic double bond suggested the presence of an isopropenyl moiety. Therefore, on the basis of NMR (^1^H, ^13^C, DEPT, HMQC and HMBC) and mass spectral data and comparison with those reported in the literatures and the structure of the compound was identified as Betulin (Lupan-3β,28-diol) having molecular formula C_30_H_50_O_2,_ Molecular weight of 442.72 and melting point of 251.6^24^. (Fig. 4).

### Biological activities of betulin

**Figure 5:**
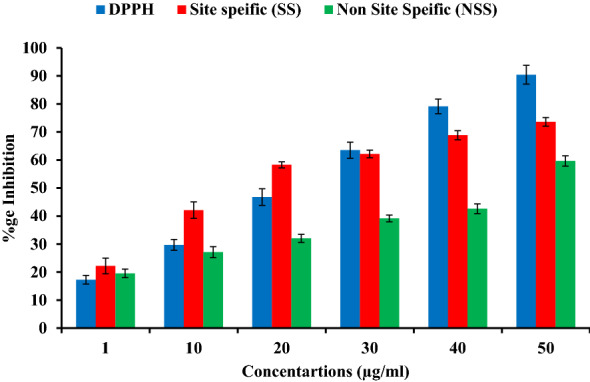
In vitro DPPH and hydroxyl radical scavenging potential of “Betulin” by site and non-site specific deoxyribose degradation assay.

Figure 5 depicts the positive dose dependent DPPH radical scavenging potential of betulin. The addition of betulin led to change in colour, with a very fast reaction speed up to a concentration of 50 µg/ml. At 50 µg/ml concentration betulin exhibited 88.67% of activity (IC_50_ 23.75 µg/ml), and there is no change in colour and inhibition potential after this concentration. These *in-vitro* DPPH radical scavenging potential of Betulin revealed remarkable antioxidant potential. Previous studies reported the antioxidant activity of plant extracts has a positive correlation with percentage radical scavenging activity^25^. Therefore, an extract with high percentage radical scavenging activity ought to be a potent antioxidant in vitro and in vivo. The high percentage radical scavenging activity translates to low EC50/IC50 values^26^.

Betulin also exhibited very good site (72.83%) & non-site (58.44%) specific hydroxyl radical scavenging potential at 50 µg/ml concentration and the results also showed that there is slight difference in the antioxidant potential of betulin in the site & non site specific modes of deoxyribose degradation assays.

In chelating power assay, betulin isolated from *A. nilotica* interfered with the formation of ferrous and ferrozine complexes, and have good chelating activity of 75.22% (IC_50_ 58.24 µg/ml) at 250 µg/ml concentration and are able to capture ferrous ion before ferrozine (Fig. 5).

**Figure 6:**
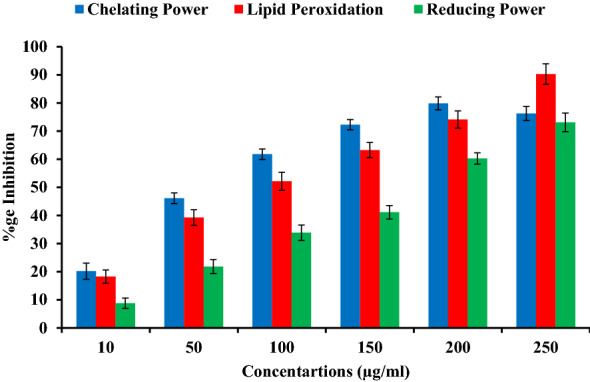
In vitro chelating power, reducing power and lipid peroxidation inhibition of “Betulin” through chelating reducing and Lipid peroxidation assays.

Figure 6 also showed the dose response ability of the betulin to reduce Fe(III) to Fe(II) at different concentrations. This reduction helps to predict the betulin ability to mimic the body’s endogenous antioxidants like bilirubin and uric acid in attenuating oxidative stress^27,28^. Therefore, high ferric reducing antioxidant power is correlated with increase in absorbance values and low IC_50_ values. Our results are confirmatory with previous reports which found that catechin, (epi) gallocatechin and caffeic acid present in the stem bark crude extract of *S. crude* have good antioxidant activities against DPPH radical scavenging and reducing power activities with low IC50 values^29^.

In lipid peroxidation assay, the betulin exhibit moderate to strong antioxidant potential i.e. 16.25–90.1567.2 ± 1.8% at 10–250 µg /ml concentration (weak–good). All the values of antioxidant activities were considered to be significant at *P* ≤ 0.05.

**Figure 7:**
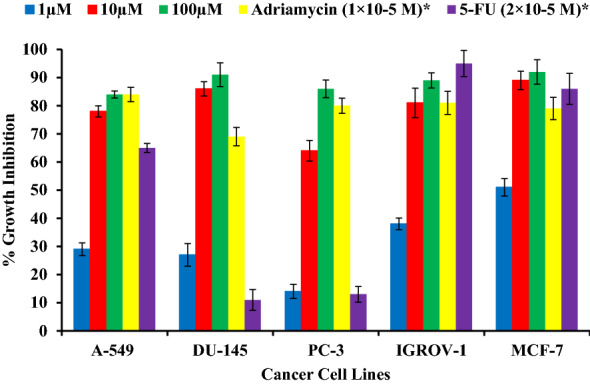
In vitro growth inhibition potential of “Betulin” against different human lung, prostate, ovary and breast cancer cell lines.

In this research work, cytotoxic activities of Betulin toward the A-549, DU-145, PC-3, IGROV-1 & MCF-7 cell lines was determined and the growth inhibition percentage by betulin is shown in Table 3 and Fig. 7. Betulin exhibited excellent potent anticarcinogenic potential at different concentrations. At 100 μM concentration, betulin exhibits 84% (A-549), 91% (DU-145), 86% (PC-3), 89% (IGROV-1) & 92% (MCF-7). Positive controls showed 84, 69, 80, 81, 79 (Adriamycin) 65, 11, 13, 95, 86 (5-FU) for A-549, DU-145, PC-3, IGROV-1 & MCF-7 cell lines at 1 × 10^–5^ M 2 × 10^–5^ M concentrations respectively.

**Table 3:** In vitro cytotoxicity activities of betulin against different human cell lines.

Compound	Concentration	A-549 (Lung)	DU-145 (Prostate)	PC-3 (Prostate)	IGROV-1 (Ovary)	MCF-7 (Breast)
Betulin	1 µM	29	27	14	38	51
	10 µM	78	86	64	81	89
	100 µM	84	91	86	89	92
Adriamycin	1 × 10^–5^ M	84	69	80	81	79
5-FU	2 × 10^–5^ M	65	11	13	95	86

Betulin isolated from *A. nilotica* was also found to be a selective inhibitor of COX-2 (COX-2 selectivity > 10). At a concentration of 10 μM, it inhibited the COX-1 by 43.81% whereas COX-2 was inhibited by 95.03% (Table 4). The ethyl acetate fraction of methanol extract also demonstrate strong capacity to suppress this inflammatory pathway. In the presence of betulin, the level of PGE_2_ dropped too low. Flavonoids and other phenolic compounds are known to target cyclooxigenase-mediated inflammation^30–32^ HPLC based presence of polyphenols and these polyphenols already reported to block cyclooxygenase activity induced by UVB radiation. Thus it might also be implicated in suppression of cyclooxygenase-mediated inflammatory pathway^33^.

**Table 4:** Cyclooxygenase enzyme mediated anti-inflammatory activities (COX-1 & COX-2) of “Betulin “isolated from bark of *Acacia nilotica.*

Compound	% Inhibition			IC_50_ (µM)		COX-2 selectivity*
	COX-2		COX-1	COX-2	COX-1	
	1 µM	10 µM	10 µM			
Betulin	56.22	95.03	43.81	< 1.0	> 10	> 10
Rofecoxib**	75	100	75	0.3	40	˜133
Celecoxib **	50	100	65	1.2	14	˜10

*COX-2 selectivity = IC_50_ (COX-1)/ IC_50_ (COX-2).

**Reported in literature (Kaur et al., 2009)^22^.

## Discussion

In the past two decades triterpenes have attracted attention because of their pharmacological potential. Among them, betulin is the most abundant and it is a representative compound of *Betula platyphylla*, a tree species belonging to the Betulaceae family^34^. Betulin has been demonstrated to have a selective cytotoxicity in tumor cell line^35–37^. It has also shown a strong reduction of hepatotoxicity^38^. Furthermore, betulin was shown to exhibit chemopreventive effects on UV induced DNA damage in *congenital naevi* (CMN) cells^39^. Previous studies on betulin also shown protective effects against Cd-induced cytotoxicity occur via the anti-apoptosis pathway in Hep3B cells, ethanol induced cytotoxicity in HepG2 and potent superoxide anion generation inhibitors in human neutrophils^40–42^. The antioxidant property of betulin was confirmed by its ability to scavenge and prevent the attack of free radicals on the membranes by increasing its negative surface charge^43^. Betulin and betulinic acid have been shown as potent phospholipase A2 inhibitors^44^. Furthermore, betulin acts as a modest TNF-α inducer by enhancing mitogen-induced TNF-α production, and betulinic acid modulates cytokine production by Th1/Th2 cell subpopulations^45^. In the present study, we have isolated betulin from ethyl acetate fraction of methanol extract of *A. nilotica* and checked their different antioxidant, cytoprotective and anti-inflammatory activities employing a battery of in vitro assays. It is important to use different assays, instead of relying on a single assay to assess and compare the antioxidant capacity.

The methanol extract showed high biological potential than the other crude extracts of the *A. nilotica* (data not shown) and these results suggested that these high biological activities might be due to the high TPC. Many previous studies observed the direct relationship between TPC and antioxidant activity in medicinal plant extracts. The phenolic compounds may contribute directly to antioxidative action or as free radical scavengers due to their hydroxyl groups^46^. Tanaka et al.^47^, reported that 1 g phenolic compounds daily from a diet rich in fruits and vegetables have inhibitory effects on mutagenesis and carcinogenesis in humans. Currently the interests of phenolic compounds are increasing in the food industry because they retard oxidative degradation of lipids and thereby improve the quality and nutritional value of food^48^.

The HPLC based phenolic fingerprinting of methanol extract of *A. nilotica* showed the presence of many phenolic components such as gallic acid, quercetin, myricetin, rutin, kaempferol, catechin, epicatechin, ferulic acid, betulin and umbelliferone and are very significant to understand the relationship between the phenolic composition and bioactivities. The enrichment of the extract with polyphenols might responsible for the potent biological activities of the extract. Several previous studies have reported that these polyphenols exhibited strong antioxidative, anticancer, and anti-inflammatory activities^49–52^.

The methanol extract of *A. nilotica* showed the highest amount of TPC (835 mg/g as GAE) which lead us for the chromatographic & spectroscopic analysis of methanol extract. The chromatographic analysis on precoated Kieselgel 60_254_ plate (0.2 mm thick; Merck, India), showed many spots of UV & iodine sensitive compounds. The repeated column chromatography of the fraction 54–156 (group 3) as shown in Fig. 2, resulted in the isolation of white amorphous powder which was positive in the Liebermann-Burchard test for triterpenoids (M^+^H]^+^ at m/z 443 & molecular formula C_30_H_50_O_2_). The NMR, Mass spectroscopy techniques and previous reports established the chemical structure of compound as Betulin (Lupan-3ß,28-diol)^24^.

In results of the present study we found that betulin was effective for reducing the stable DPPH radical to the yellow colored diphenylpicryl hydrazine, indicating their DPPH radical scavenging potential. The higher DPPH radical scavenging potential and lower IC_50_ values are inverse to each other. It is pertinent to mention here that, DPPH potential may be due to the hydrogen atom donating ability of betulin, which further help in trapping free radicals. EDTA is used as the metal chelator in chelating power assay as it is a strong metal chelator. In the present study, betulin exhibits good reducing potential (Fig. 6). Natural plants/extracts having chelating potential are believed to inhibit lipid peroxidation by stabilizing transition metals^53^. In reducing power assay, the yellow colour of the test solution changes to various shades of green and blue based upon the reducing power of the tested compound. The reductive ability assay suggests that the betulin is able to donate electron, hence they should be able to donate electrons to free radicals in actual biological or food systems, making the radicals stable and unreactive. Reducing power is one mechanism of action of antioxidants and may serve as a significant indicator of potential antioxidant activity^54^. Previous reports also found dose dependent manner of hydroxyl radicals scavenging potential of xylose and lysine Maillard reaction products^55^. The free radical scavenging capability of phenolics are closely related with structural formation, molecular weight and presence of aromatic rings & hydroxyl groups of the phenolics^56^.

Recent reports found that betulin to be active against colorectal, breast, prostate & lung cancer cell lines^57,58^. Betulin is a natural compound, which contains derivatives that have been shown to possess strong anti-tumor properties^5,59^. Recent studies also found that betulin in combination with cholesterol, is a very potent agent in killing cancer cells *in vitro*^60^.

Inflammation is a complex process, which involves many cell signaling pathways in addition to free radical production which are responsible for tissue degeneration and many diseases viz. rheumatoid arthritis, arteriosclerosis, myocarditis, infections, cancer, metabolic disorders^61–63^. COX-2 is an enzyme which is necessary for the production of pro-inflammatory prostaglandins and thus has been a target for many present anti-inflammatory and cancer-preventive drugs^64^. Several natural products of plant origin have been shown to transmit their anti-inflammatory activities through suppression of COX-2^65–67^.
